# Novel missense *ALDH18A1* variant in a family with autosomal dominant spastic paraplegia

**DOI:** 10.1007/s00415-025-13444-y

**Published:** 2025-12-04

**Authors:** Federica Novarella, Alessandra Tessa, Chiara Criscuolo, Gianmaria Senerchia, Valerio Nicolella, Rosanna Trovato, Fabrizia Falco, Sirio Cocozza, Alessandra Scaravilli, Margherita Ruoppolo, Marianna Caterino, Daniela Terracciano, Giuseppe Castaldo, Vincenzo Brescia Morra, Filippo Maria Santorelli, Marcello Moccia

**Affiliations:** 1 Department of Neuroscience, Reproductive Science and Odontostomatology, Federico II University of Naples, Naples, Italy; 2 Department of Molecular Medicine, IRCCS Stella Maris Foundation, Pisa, Italy; 3 Neuroradiology Unit, Policlinico Federico II University Hospital, Naples, Italy; 4 Department of Advanced Biomedical Sciences, Federico II University of Naples, Naples, Italy; 5 Department of Molecular Medicine and Medical Biotechnology, Federico II University of Naples, Naples, Italy; 6 CEINGE Biotecnologie Avanzate -Franco Salvatore, Naples, Italy; 7 Department of Translational Medical Sciences, Federico II University of Naples, Naples, Italy

**Keywords:** hereditary spastic paraplegia, ALDH18A1, genetic, neurophysiology, biomarker

## Abstract

**Objectives:**

We aim to characterize a novel heterozygous missense variant c.1703A > G/p. (Gln568Arg) in the *ALDH18A1* gene, identified in a family with autosomal dominant hereditary spastic paraplegia (HSP). We evaluated clinical, neurophysiological, genetic, fluid biomarkers, and neuroimaging expression in different family members with and without the mutation.

**Methods:**

A comprehensive multimodal evaluation was performed, including neurological examination, motor and sensory conduction studies, transcranial magnetic stimulation, targeted genetic sequencing, biomarker analysis (amino acid profiling and plasma neurofilament light chain levels (pNfL)), and brain and spinal cord MRI. Mutation pathogenicity was assessed using in silico prediction tools and confirmed by segregation analysis in family members.

**Results:**

The index case, a 53-year-old male, presented with progressive bladder and gait disturbances, with spasticity, weakness, brisk reflexes and reduced deep sensation in the lower limbs. Neurophysiological findings confirmed corticospinal tract involvement. MRI showed selective cerebellar atrophy. Genetic analysis identified the c.1703A > G/p. (Gln568Arg) mutation in the index case and in his father, presenting with similar clinical expression. pNfL was numerically higher in mutation carriers than the healthy brother and daughter of the index case, indicating neuro-axonal damage. Amino acid profiling showed normal levels of ornithine, citrulline, arginine, and proline.

**Conclusion:**

This study expands the spectrum of *ALDH18A1*-associated HSP, highlighting a novel mutation with a relatively late-onset and mild clinical phenotype. Further functional studies and longitudinal assessments are needed to confirm the pathogenicity of this variant and its potential implications for disease progression and therapeutic strategies.

**Supplementary Information:**

The online version contains supplementary material available at 10.1007/s00415-025-13444-y.

## Introduction

Hereditary spastic paraplegias (HSPs) are a group of rare neurodegenerative conditions that predominantly involve the corticospinal tract with marked paraparesis [1, 2]. Clinically, HSPs can be pure if characterized by gradually worsening pyramidal syndrome (e.g., weakness and spasticity in the lower limbs, signs of corticospinal tract involvement, as well as variable urinary disturbances), or complex if combined with additional complications (e.g., neurological, including ataxia, extrapyramidal symptoms, peripheral neuropathy, and cognitive changes; or non-neurological, including gastroesophageal reflux, Dupuytren’s disease, cataracts or varicose veins) [3]. To date, different HSP subtypes have been identified, and are characterized by a wide genetic heterogeneity (autosomal dominant, autosomal recessive, or X-linked inheritance patterns), with the complex forms being more frequently associated with recessive mutations [4]. This genetic diversity underscores the complex molecular basis of spastic paraplegias (SPG), involving mutations in numerous genes with roles in various cellular processes [5].

Pathogenic variants in *ALDH18A1* have been associated with both recessive and dominant forms of SPG9 (SPG9A and SPG9B, respectively). The *ALDH18A1* gene encodes P5CS (Δ1-pyrroline-5-carboxylate synthetase), a bifunctional enzyme involved in the urea cycle and amino acid biosynthesis. Pathogenic variants in *ALDH18A1* impair P5CS activity, potentially reducing the de novo production of ornithine, citrulline, arginine, and proline. Dominant mutations appear to exert their effects via a dominant-negative mechanism, impairing P5CS functionality, while recessive mutations lead to a loss or significant reduction of enzymatic activity [6]. These mutations are also implicated in other neurocutaneous syndromes, such as autosomal recessive cutis laxa (ARCL3A) [7]. In this article, we describe a family in which a novel missense variant in *ALDH18A1* has been identified, thus expanding the molecular spectrum of HSPs and related clinical expression.

## Methods

Following the identification of the novel variant in the index case, a comprehensive multimodal evaluation was performed in symptomatic and asymptomatic family members, including genetic testing and other procedures, aimed at minimizing any potential adverse consequences. All subjects gave a written informed consent to genetic testing and other study procedures, and to the publication of anonymized results. The local Ethical Committee approved this study.

### Clinical and neurophysiology studies

The index case was seen at the Neurology Unit of the Policlinico Federico II University Hospital in Naples, Italy.

Based on clinical history and neurological examination, the index case was referred to neurophysiology studies, including motor conduction velocities (MCV) and sensory conduction velocities (SCV) (which were evaluated on the right median nerve, left ulnar nerve, right posterior tibial nerve, right deep peroneal nerve, right sural nerve, right superficial peroneal nerve, and right anterior tibial nerve). Transcranial magnetic stimulation was assessed at the level of the left orbicularis oris muscle, right and left dorsal interosseous muscles, and right and left abductor hallucis muscles.

We explored cognitive function using a neuropsychological battery including the mini mental status examination (MMSE), along with more specific tests for different cognitive domains (e.g., memory, attention, executive, language, visuospatial), neuropsychiatric features (neuropsychiatric inventory questionnaire), activities of daily living (ADL), and instrumental ADL (iADL). Diagnosis of MCI was based on the scoring ≥ 1.5 standard in ≥ 1 cognitive domain in the absence of impairments in activities of daily living.

Following this comprehensive clinical assessment, the index case was referred to genetic studies and, based on genetic results, family members were recruited.

### MRI studies

Brain and spinal cord images of the index case were acquired on a 1.5 Tesla MR scanner (Sola, Siemens Medical Systems, Erlangen, Germany). Brain protocol included a volumetric T1-weighted sequence (Magnetization Prepared Rapid Gradient Echo—MPRAGE; TR = 2200 ms; TE = 2.6 ms; TI = 900 ms; flip angle = 8°; voxel size = 1 × 1x1 mm^3^; number of sagittal slices = 192), a volumetric Fluid Attenuated Inversion Recovery (FLAIR) sequence (TR = 6600 ms; TE = 380 ms; TI = 1800 ms; voxel size = 1 × 1x1 mm^3^; number of sagittal slices = 176), and a Susceptibility Weighted Imaging (SWI) sequence (TR = 49 ms; TE = 40 ms; voxel size = 0.8 × 1.2x2 mm^3^; number of axial slices = 80).

### Genetic studies

DNA samples from recruited family members were isolated from whole blood using standard procedures [8]. Participants in the present study provided informed consent for DNA storage and granted permission for the use of this material in current diagnostic and research procedures. The list of diseases, genes, and techniques that were included in the analysis is reported in Supplementary Material 1.

### Biomarker studies

Blood samples were collected simultaneously in BD Vacutainer^™^ tubes containing EDTA as an anticoagulant, centrifuged within 3 h at 1100 rpm for 10 min, aliquoted into polypropylene tubes, and stored at − 80 °C.

Plasma amino acid concentrations were analyzed using HPLC with an Agilent Technologies 1200 Series LC system, equipped with an Agilent Zorbax Eclipse XDB-C18 analytical column (5 µm, 4.6 × 150 mm) and an Agilent Eclipse XDB-C18 guard column (5 µm, 4.6 × 12.5 mm). Plasma aliquots of 500 µL were examined. Derivatization of sample was performed using o-phthalaldehyde (OPA) for amino acids with primary ammine and 9-fluorenylmethyl chloroformate (FMOC) for amino acids with secondary amine group. The chromatographic separation was achieved using: flow rate 1.3 mL/min; temperature 40 °C; solvent A, 40 mM phosphate buffer, pH 7.8 and solvent B, CH₃CN/CH₃OH/H₂O, 40/40/20. The linear elution gradient followed the subsequent method: 10–20% of B in 6 min; 20–27% of B in 6 min; 27–60% of B in 10 min; 60–100% in 2 min; final isocratic step at 100% of B for 6 min. Amino acids were identified based on their retention times and quantified by absorption ratio, comparing them with the ratio of authentic standards in the calibration solution, which contained a mixture of amino acids at a final concentration of 200 µM.

In addition, plasma neurofilament (pNfL) levels were measured in all four family members, in a single session, using Lumipulse^™^ assay kits (Fujirebio, Tokyo, Japan) on the Lumipulse G system, a chemiluminescent enzyme immunoassay (CLEIA) analyzer. For the interpretation of pNfL, age-derived percentiles were calculated using a previously validate tool [9, 10].

## Results

**Fig. 1:**
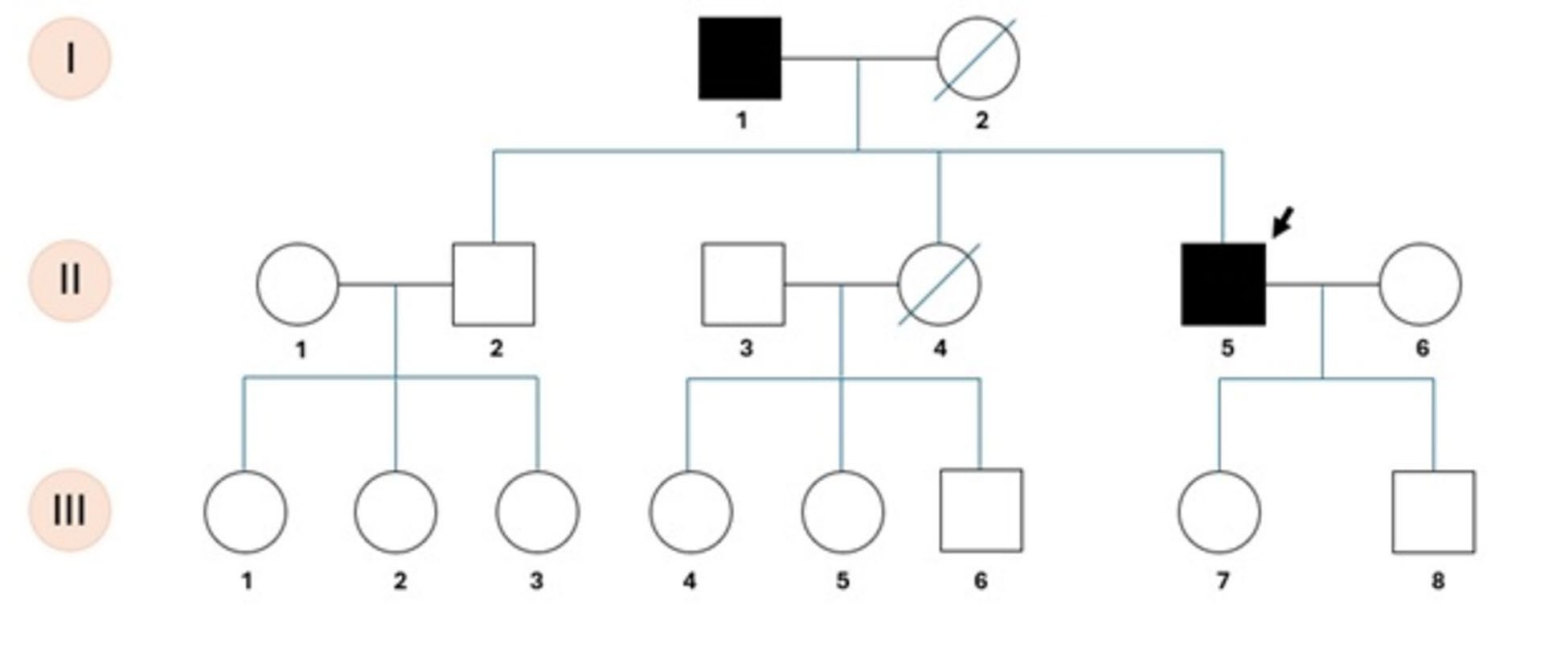
Pedigree of the family. The index subject is indicated by the arrow (II-5). The other subjects who underwent genetic analysis are I-1, II-2, and III-7. Other subjects potentially carrying the mutation did not consent to testing

Summary of clinical, neurophysiology, genetic, biomarker and MRI results is provided in Table 1. Pedigree of the family is presented in Fig. 1.

**Table 1:** Clinical, neurophysiology, genetic, biomarker, and MRI results

	Heterozygous c.1703A > G/p.Gln568Arg variant in ALDH18A1		No mutation	
	I-1	II-5	II-2	III-7
Clinical			Normal	Normal
Age at onset	60 s	51	–	–
Symptoms at onset	Unsteadiness	Gait disturbances	–	–
Age at examination	91	53	59	23
Spastic paraparesis	Mild spasticity and mild weakness in the lower limbs	Mild spasticity, mild weakness and brisk reflexes in the lower limbs	–	–
Plantar response	Absent	Absent	Flexor	Flexor
Deep sensation	Reduced in the lower limbs	Reduced in the lower limbs	Normal	Normal
Bladder dysfunction	Frequency and urgency	Frequency and urgency	–	–
Cerebellar	Broad-base gait, slow saccades	Broad-base gait, scanning speech, nystagmus, slow saccades	Normal	Normal
Comorbidities	Hypertension, diabetes	None	None	Gastroesophageal reflux
Neurophysiology	Not tested	Corticospinal pathway damage to lower limbs, normal corticobulbar and upper limb pathways	Not tested	Not tested
Cognitive function	Amnestic multi-domain mild cognitive impairment	Normal	Normal	Normal
Amino acids	↑ Arginine, cystine, sarcosine, proline	↑ Glycine, alanine, isoleucine	↑ Sarcosine	Within normal ranges
Plasma NfL (pg/mL)	17.07	7.59	6.70	4.28
Plasma NfL percentile	n/a	50-75th percentile	10-25th percentile	25-50th percentile
MRI	Not available	Selective cerebellar tonsil atrophy, enlargement of retrotonsillar fissure	Not available	Not available

### Clinical and neurophysiology results

A 53-year-old man (index case, II-5) presented to our clinic with gait disturbances that had developed over the previous 2 years. He was otherwise healthy (i.e., no involvement of systems other than neurological). Clinical examination revealed spastic paraparesis (mild spasticity, mild weakness and brisk reflexes in the lower limbs, with no plantar response), reduced deep sensation in the lower limbs, bladder dysfunction (with urinary frequency and urgency), broad-base spastic gait (that worsens with eyes closed), slow saccades, and scanning speech.

Motor evoked potentials revealed damage to the corticospinal tracts of the lower limbs, while the corticobulbar tract to the orbicularis oris muscle and the corticospinal tracts of the upper limbs were within normal limits. In addition, nerve conduction studies showed no neurophysiological signs of peripheral neuropathy.

Looking at family members, subject I-1 (father of the proband) is a 91-year-old individual with hypertension, diabetes, and amnestic multi-domain mild cognitive impairment (MCI); on clinical examination, we found spastic paraparesis (mild spasticity and mild weakness in the lower limbs, with no plantar response), reduced deep sensation in the lower limbs, bladder dysfunction (with urinary frequency and urgency), and broad-base spastic gait (that worsens with eyes closed), slow saccades, and nystagmus. Case II-2, the 59-year-old healthy brother of our index case, and case III-7, the 23-year-old daughter of the index case, had normal neurological examination, though the latter had recently experienced multiple episodes of gastroesophageal reflux.

### MRI results

**Fig. 2:**
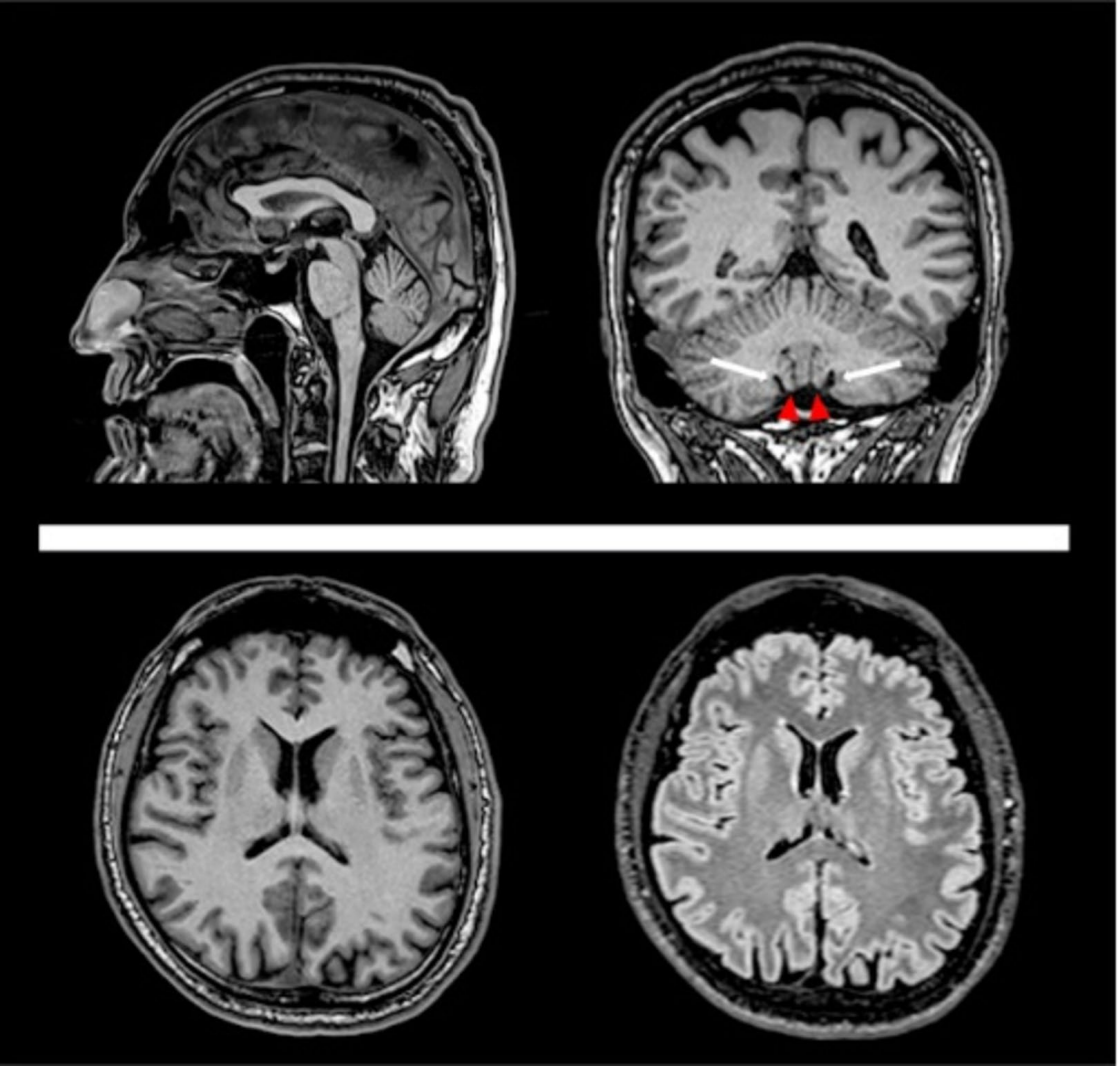
Brain MRI. Representative images of the brain magnetic resonance imaging (MRI) scan in the index case (II-5). In the upper row, a sagittal slice of a 3D volumetric T1-weighted image on the left shows the absence of significant brainstem or vermian atrophy, coupled to a preserved volume of the corpus callosum. The corresponding coronal multi-planar reconstruction on the right shows the occurrence of a selective atrophy of the cerebellar tonsils (red arrowheads), confirmed by a selective enlargement of the retrotonsillar fissure (white arrows) in the context of a preserved cerebellar volume. In the lower row, two axial multi-planar reconstructions of a 3D volumetric T1-weighted image (left) and a 3D Fluid Attenuated Inversion Recovery (FLAIR) sequence (right) show the absence of significant brain atrophy and signal changes, respectively

Brain MRI of the index case (II-5) showed a peculiar pattern of atrophy affecting the cerebellar tonsils (depicted by a selective enlargement of the retrotonsillar fissure) within the context of a globally preserved cerebellar volume (Fig. 2), in the absence of other ill-defined changes. Spinal cord was within normal limits.

### Genetic results

**Fig. 3:**
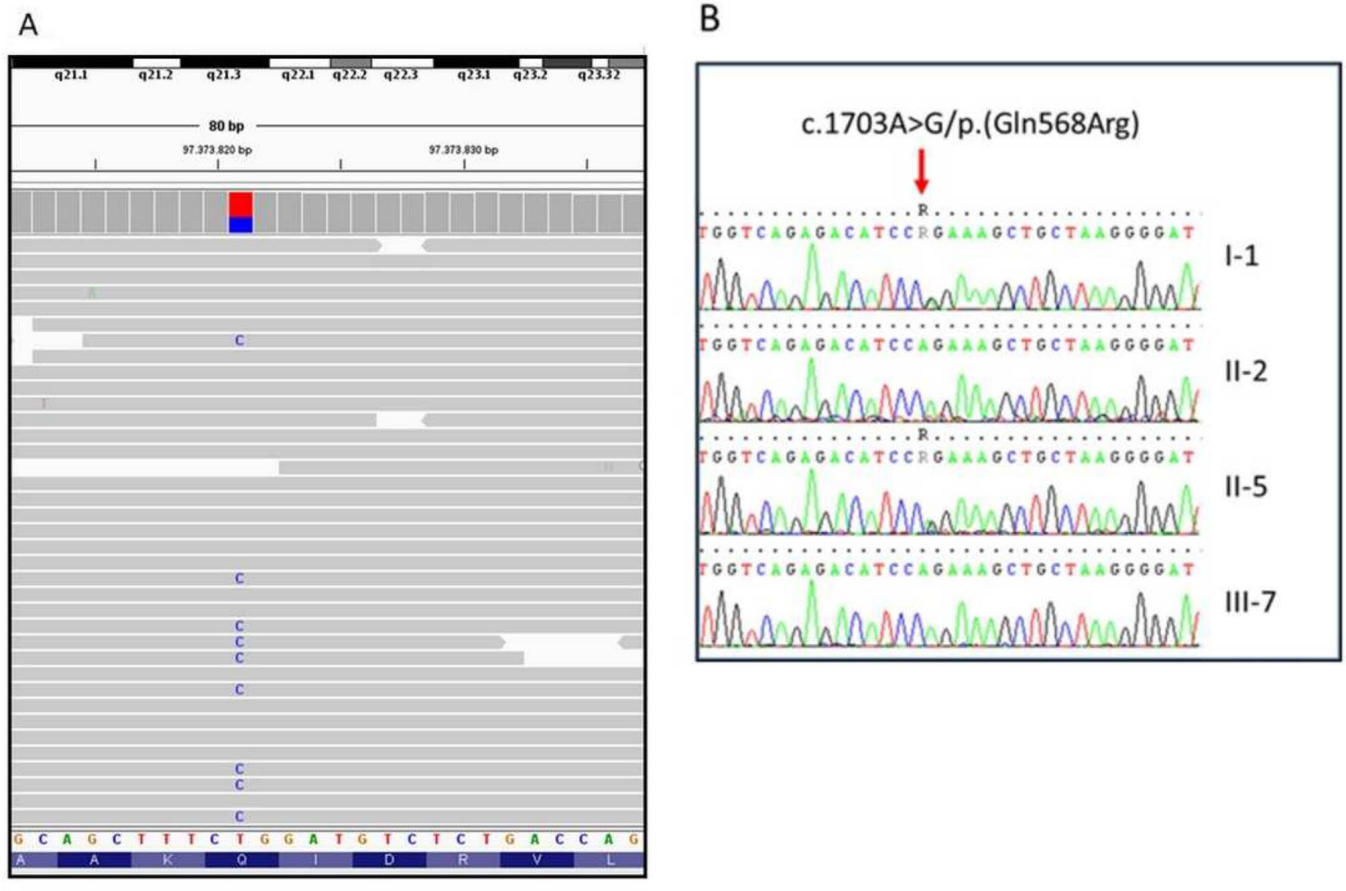
NGS sequencing reads and electropherograms. NGS sequencing reads is visualized with Integrative Genomic Viewer (IGV) for the heterozygous c.1703A > G/p.(Gln568Arg) variant in exon 14 of ALDH18A1 of proband II-5 (reverse strand) (**A**). Electropherograms is obtained from Sanger sequencing of the region flanking the c.1703A > G variant in ALDH18A1 (NM_002860.4) in proband II-5 and in his family members; the arrow indicates the heterozygous variant in I-1 and II-5 (**B**)

This analysis revealed in the proband the heterozygous c.1703A > G/p.(Gln568Arg) variant in the *ALDH18A1* gene (NM_002860.4), validated by Sanger sequencing (Fig. 3).

The heterozygous c.1703A > G/p. (Gln568Arg) had a low frequency (0.00002) in gnomAD [11], a CADD score of 22.4, and a REVEL Score of 0.45 [12]. The variant had ambiguous interpretations over multiple predictive bioinformatic tools. It was predictably pathogenic in FATHMM-MKL and in Mutation taster, while it scored as of uncertain significance in 12 in silico tools. The variant is classified as “uncertain significance” (criteria PM2) according to the current guidelines of the ACMG. Segregation analysis showed the presence in the affected father (case I-1), but not in healthy brother (case II-2) and healthy daughter (case III-7).

### Fluid biomarker results

The proband exhibited slightly elevated levels of glycine, alanine, and isoleucine, in respect to reference range. However, the levels of amino acids directly involved in the activity of the protein product of the mutated gene—ornithine, citrulline, arginine, and proline—were within normal ranges. Similarly, in the other carrier of the mutation (I-1), we observed an increase in arginine, cystine, sarcosine, and proline, but normal levels of ornithine, citrulline, arginine, and proline. The values of III-7 were within the reference range, while in II-2, only a slight increase in sarcosine was observed.

Higher plasma NfL levels were found in mutation carriers I-1 (17.07 pg/mL) and II-5 (7.59 pg/mL), when compared with healthy family members (eldest brother of the proband (II-2 6.70 pg/mL) and his daughter (III-7 4.28 pg/mL)) (Table 1).

## Discussion

We have shown a family with autosomal dominant HSP carrying a novel missense variant in *ALDH18A1*. The presence of HSP on neurological examination was confirmed by electrophysiological studies, and brain and spinal cord MRI also ruled out other possible conditions. The novel mutation is probably disease causative because the uncertainty derived from multiple prediction bioinformatic tools is mitigated by the family segregation studies where core clinical features (spastic paraparesis, bladder dysfunction and reduced deep sensation in the lower limbs)[13], and with higher levels of NfL, as from ongoing neuro-axonal damage[14].

In keeping with this, mutation carriers presented with spastic paraparesis (mild spasticity, mild weakness and brisk reflexes in the lower limbs, with no plantar response), reduced deep sensation in the lower limbs, and bladder dysfunction (with urinary frequency and urgency), that are the typical clinical features of HSP and have also been described in SPG9B families (Table 2) [13, 15]. However, the two carriers of the variant had a complex clinical presentation, with evidence of cerebellar involvement on clinical examination (broad-base gait and scanning speech) and on brain MRI. Notably, compared to other previously reported SPG9B families [16] (Table 2), our case had a later disease onset, occurring around 50 years of age. Unlike some SPG9A cases [17] (Table 2), where patients exhibited also changes in the upper limbs, our proband had exclusive involvement of the lower limbs on both clinical examination and neurophysiology testing.

**Table 2:** Comparison between families with SPG9B

Reference	Novarella et al. (2025)	Coutelier et al. (2015)			Marelli et al. (2020)
ALDH18A1 mutation	p.Gln568Arg	p.Val120Ala	p.Arg665Leu	p.Arg252Gln	p.Ala224Ser
Number of described individuals	2	7	4	3	4
Age at onset	51–60	14–59	13–42	1–43	5–50
Neurological examination	Complex form with spastic paraparesis and cerebellar signs	Complex form with clinical variability (spastic dysarthria, cerebellar signs, dementia)	Spastic gait with pyramidal signs	Spasticity	Considerable variability from severe infantile forms to mild forms with only spasticity
Neurophysiology	Corticospinal pathway damage to lower limbs	Motor neuropathy	Motor neuropathy	Not available	Normal
Brain MRI	Selective cerebellar tonsil atrophy, enlargement of retrotonsillar fissure	Normal or mild corpus callosum atrophy	Inter-individual variability: white matter abnormalities, increased pontine signal, spinal cord thinning, enlarged cisterna magna	Not available	Mild spinal atrophy, mild corpus callosum hypoplasia
Amino acids	Mild, non-specific increase in various amino acids	Low levels of citrulline, proline, ornithine, and borderline levels of arginine	One individual with very low citrulline levels, low arginine levels, and borderline ornithine levels	Not available	Low citrulline levels
Family code		FSP410	FSP429	FSP470	

NfL, a well-established biomarker of neuro-axonal damage, is expected to increase in the context of HSP, though not as much as in other neurodegenerative disease [14]. In our family members with missense variant of *ALDH18A1*, pNfL was consistently higher than in healthy family members (absolute values and age-derived percentiles), thus suggesting a correlation between the presence of the c.1703A > G/p.Gln568Arg mutation in ALDH18A1 and neurodegenerative changes. Notably, despite being older, II-2 (who does not carry the mutation) exhibited lower NfL levels than his younger, mutation-positive sibling (II-5), further supporting the pathogenic role of this variant. Interestingly, age is expected to be a main predictor of NfL levels[18], while in our family, the elder brother (not mutated) had lower levels than the younger brother (mutated), further suggesting a pathogenetic role of the variant.

In the proband, we also found a relatively peculiar brain MRI pattern of atrophy, with a selective involvement of cerebellar tonsil. This finding is in line with the complex clinical presentation (including cerebellar symptoms) and respective neuroradiological complexity [19, 20]. Considering that specific patterns of cerebellar atrophy are reported in other HSPs (for instance, a prominent involvement of superior vermis in SPG7 [21]), the pattern of atrophy of the proband might reflect the complex presentation of SPG9B, although this speculation warrants further investigation.

This case underscores the continuous need to explore the molecular complexity of HSPs and its clinical implications. The coexistence of distinct clinical syndromes with dominant or recessive inheritance patterns highlights the complexity of these disorders. It was proposed a unifying disease model based on a continuum of increasing severity, ranging from SPG9A to SPG9B, ADCL3, and ARCL3A. This continuum reflects the progressive disruption of P5CS activity, driven by mechanisms such as loss of function or dominant-negative effects [22]. Our mutation likely causes a relatively milder clinical phenotype, compared with previously described families (Table 2), as suggested by the older age at onset. Accordingly, while decreased plasma levels of ornithine, citrulline, arginine, and proline have been observed in *ALDH18A1*-related syndromes [23], we observed normal levels of these amino acids. Interestingly, we saw abnormally high levels of other amino acids in both mutation carriers, that are unlikely related to our mutation (and clinical symptoms) and could likely reflect dietary intake and tissue catabolism [22]. Interestingly, families with SPG9B and earlier age at onset also had lower levels of these amino acids [7]. In our case, ornithine, citrulline, arginine, and proline were normal in the peripheral blood, but this does not imply their levels were normal within the central nervous system. Indeed, it is possible that amino acid production was impaired in areas with higher metabolic needs (e.g., corticospinal tract), as in the case of some families with HSP presentation during pregnancy, when there are higher metabolic demands [24].

We have to acknowledge a number of limitations. This study is based on a single family, which limits the generalizability of the findings. Additional cases (including other family members and independent families) would be needed to determine whether this mutation consistently results in the suggested milder clinical phenotype. While in silico analysis predicted the mutation as being in intolerant position, no functional studies were conducted to confirm its impact on P5CS activity; in particular, biochemical and cellular assays could provide further validation. We based, at least in part, our conclusions on the levels of pNfL, but their interpretation holds limitations in the absence of well-established cut-offs [25]. Longitudinal data are currently lacking in SPG9, preventing an assessment of disease progression (i.e., late-onset cases might remain relatively stable over time).

In conclusion, our results contribute to the understanding of the clinical variability of *ALDH18A1*-related HSP. We have described a family with complex presentation of HSP, based on clinical, neurophysiology, and MRI results, and novel variant in *ALDH18A1*, with autosomal dominant inheritance. We have leveraged segregation analysis and NfL levels to confirm this association, but further functional studies are needed to understand the biological mechanisms.

## Supplementary Information

Below is the link to the electronic supplementary material.

Supplementary file1 (DOCX 31 KB)

## Data Availability

Data is available upon request to the corresponding author.
