# Supplementary material for: Novel missense ALDH18A1 variant in a family with autosomal dominant spastic paraplegia
Source: J Neurol. 2025 Dec 4;273(1):6. doi: 10.1007/s00415-025-13444-y (PMC12678542; doi:10.1007/s00415-025-13444-y)
Supplement: Supplementary file 1 — Supplementary file1 (DOCX 31 KB) [file 415_2025_13444_MOESM1_ESM.docx]

# Supplementary Material 1. Full genetic panel.

Table shows the list of diseases, genes and techniques that were included in the analysis.

| **Disease** | **Gene** | **Analysis technique** |
| --- | --- | --- |
| SCA1 | *ATXN1* | TP-PCR method with the SCAs kit-FL (Experteam) for SCA1-2-3-6-7. In house TP-PCR method for SCA8-12-17.  All analyses were performed according to the best practice guidelines for SCA analysis. Alleles were classified as normal, intermediate (if relevant) and pathogenic, according to established ranges (https://www.ncbi.nlm.nih.gov/books/NBK1138/). |
| SCA2 | *ATXN2* |  |
| SCA3 | *ATXN3* |  |
| SCA6 | *CACNA1A* |  |
| SCA7 | *ATXN7* |  |
| SCA8 | *ATXN8* |  |
| SCA12 | *PPP2R2B* |  |
| SCA17 | *TBP* |  |
| HSP NGS panel | *AARS2, ABCB7, ABCD1, ABHD12, ACO2, ADCK3, ADGRG1, AFG3L2, AHDC1, AHI1, ALDH5A1, ALG3, ALG6, AMACR, AMPD2, ANO10, APOB, APTX, ARL13B, ARSA, ATCAY, ATG5, ATL1, ATM, ATP13A2, ATP1A2, ATP1A3, ATP2B3, ATP7B, ATP8A2, BEAN1, BRAT1, BRF1, C10ORF2, C12ORF65, C19ORF12, C5ORF42, C9ORF72, CA8, CACNA1A, CACNA1G, CACNB4, CAMTA1, CASK, CC2D2A, CCDC88C, CD40LG, CDK5, CEP104, CEP290, CEP41, CHMP1A, CLCN2, CLN5, CLN6, CLN8, CLP1, COA7, COQ2, COQ4, COQ9, COX20, CP, CSPP1, CSTB, CTBP1, CTSD, CWF19L1, CYP27A1, CYP7B1, DAB1, DARS, DARS2, DDHD2, DKC1, DNAJC19, DNAJC3, DNMT1, EEF2, EIF2B1, EIF2B2, EIF2B3, EIF2B4, EIF2B5, ELOVL4, ELOVL5, ERCC4, ERCC8, EXOSC3, EXOSC8, FA2H, FARS2, FASTKD2, FAT2, FGF14, FLVCR1, FMR1, FOLR1, FXN, GALC, GAN, GBA2, GBE1, GFAP, GJB1, GJC2, GLB1, GOSR2, GRID2, GRM1, HARS, HARS2, HEXA, HEXB, HIBCH, HSD17B4, INPP5E, ITPR1, KCNA1, KCNA2, KCNC1, KCNC3, KCND3, KCNJ10, KCNMA1, KCTD7, KIF1A, KIF1C, KIF7, LAMA1, LMNB2, LYST, MARS2, MED17, MFN2, MFSD8, MKS1, MMACHC, MME, MRE11A, MTPAP, MTTP, MVK, NAGLU, NDUFS1, NDUFS7, NEU1, NOL3, NOP56, NPC1, NPC2, NPHP1, OFD1, OPA1, OPA3, OPHN1, PAX6, PCLO, PCNA, PDE6D, PDHA1, PDSS1, PDSS2, PDYN, PEX10, PEX16, PEX2, PEX6, PEX7, PHYH, PIK3R5, PLA2G6, PLD3, PLP1, PMM2, PMPCA, PNKP, PNPLA6, POLG, POLR3A, POLR3B, PPT1, PRICKLE1, PRKCG, PRNP, PRPS1, PRRT2, PSAP, PSEN1, PTF1A, PTRH2, PUM1, QARS, RAB3GAP1, RARS, RARS2, RELN, RNF170, RNF216, RPGRIP1L, RUBCN, SACS, SAMD9L, SARS, SCN1A, SCN2A, SCN8A, SCYL1, SEPSECS, SETX, SIL1, SLC17A5, SLC1A3, SLC25A46, SLC2A1, SLC33A1, SLC35A2, SLC52A2, SLC6A19, SLC9A1, SLC9A6, SMPD1, SNAP25, SNX14, SPAST, SPG11, SPG7, SPTAN1, SPTBN2, SRD5A3, STS, STUB1, STXBP1, SURF1, SYNE1, SYNE2, SYT14, TBC1D23, TCTN1, TCTN2, TCTN3, TDP1, TDP2, TGM6, TINF2, TMEM138, TMEM216, TMEM231, TMEM237, TMEM240, TMEM67, TOE1, TOP1, TPP1, TRNT1, TRPC3, TSEN15, TSEN2, TSEN34, TSEN54, TTBK2, TTC19, TTC21B, TTPA, TUBB3, TUBB4A, UBA5, UBR4, UCHL1, VAMP1, VARS2, VLDLR, VPS53, VRK1, VWA3B, WDR73, WDR81, WFS1, WWOX, XPA, XRCC1, XRCC4, ZFYVE26, ZFYVE27, ZNF423* | Illumina NextSeq500 platform (Illumina Inc., San Diego, CA). The probes used for the experiment were designed based on SureSelect technology, employing bridge PCR hybridization (Agilent Technologies, Santa Clara, CA).  The VCF file was annotated using the Expert Variant Interpreter (eVAI v2.8) software. The interpretation of variants identified in this analysis is based on current knowledge and the ACMG classification guidelines (<https://www.acmg.net/>).  The following databases were also consulted, all on 12 March 2025, for classification and interpretation of variants: the American College of Medical Genetics guidelines (https://www.acmg.net/), ClinVar (https://www.ncbi.nlm.nih.gov/clinvar/), Franklin by Genoox (https://franklin.genoox.com/clinical-db/home), Mobi Details (https://mobidetails.iurc.montp.inserm.fr/MD/), and VarSome (https://varsome.com/). |
